# Analytical sequence to study G-CSF effect on the transcriptome of isolated spinal motoneurons from SOD1 G93A mice, an animal model for amyotrophic lateral sclerosis

**DOI:** 10.1016/j.gdata.2015.02.003

**Published:** 2015-02-28

**Authors:** Alexandre Henriques, Stefan Kastner, Oliver Wafzig, Jose-Luis Gonzalez De Aguilar, Armin Schneider

**Affiliations:** a INSERM, U1118, Mécanismes Centraux et Péripheriques de la Neurodégénérescence, Strasbourg, France; b UMRS1118, Fédération de Médecine Translationnelle de Strasbourg, Université de Strasbourg, France; c Sygnis Bioscience GmbH & Co KG, Im Neuenheimer Feld 515, 69120 Heidelberg, Germany

**Keywords:** Motor neuron, ALS, G-CSF, Gene expression, Laser microdissection

## Abstract

Granulocyte-colony stimulating factor (G-CSF) has been recently identified as a neurotrophic factor able to preserve motor functions, rescue motor units and extent survival in an animal model of amyotrophic lateral sclerosis, the SOD1 G93A mice. To gain insight into the mode of action of G-CSF, we have recently performed gene expression profiling on isolated lumbar motoneurons from SOD1G93A mice, and shown that G-CSF re-adjusted gene expression in motoneurons of symptomatic SOD1G93A mice and modulates genes related to neuromuscular function (Henriques et al., 2015). Here, we provide quality controls for the microarray experiment (GO accession number GSE60856) and describe the experimental strategy.

**Table:** 

Specifications	
Organism/cell line/tissue	*Mus musculus*, laser capture spinal motoneurons
Sex	Females
Sequencer or array type	Affymetrix, GeneChip® mouse genome 430 2.0 array
Data format	Raw data (cel files)
Experimental factors	Isolated motoneurons from SOD1 G93A mice with or without pharmacological treatment based on G-CSF.
Experimental features	Combined laser microdissection, gene expression profiling to study motoneuron specific expression in an animal model of ALS, with and without neuroprotective treatment based on G-CSF.
Consent	N/A
Sample source location	N/A

## Direct link to deposited data

Deposited data can be found at: http://www.ncbi.nlm.nih.gov/geo/query/acc.cgi?acc=GSE60856.

## Experimental design, materials and methods

Amyotrophic lateral sclerosis (ALS) is a neurodegenerative disease characterized by the death of cortical, bulbar and spinal motoneurons. G-CSF is a hematopoietic growth factor with neuroprotective properties for motoneurons [2], [3]. Many studies have undertaken the study of the transcriptome of tissues or isolated cells from animal models or ALS patients [4]. The aim of the original study was to identify for the first time the transcriptional changes in motoneurons from SOD1 G93A mice upon G-CSF treatment [1].

### Mice and treatment

Two cohorts of mice have been used within the study. A first cohort consisted of SOD1G93A mice and non-transgenic littermates of 11 weeks of age. These mice were used to detect early transcriptional response due to the SOD1 G93A transgene. The second cohort of mice consisted of SOD1 G93A and non-transgenic mice receiving the vehicle solution (250 mM sorbitol, 0.004% Tween-80 and 10 mM sodium acetate buffer (pH 4)), along with a group of SOD1 G93A mice receiving G-CSF (30 μg/kg/day). This cohort was included in order to detect transcripts deregulated by ALS and transcripts modulated by G-CSF.

The administration of vehicle or treatment was achieved by osmotic pumps, from week 11 to week 15. After the sacrifice, lumbar spinal cords were dissected and snap frozen.

### Laser-microdissection and microarray

Special care was taken to avoid contamination with RNases. We have dissected 300 motor neurons per mouse using the Leica Laser Microdissection technology. Total RNA was extracted and amplified. Microarray was performed using the GeneChip® mouse genome 430 2.0 array from Affymetrix. Intensities were normalized using the Expression Console software from Affymetrix (http://www.affymetrix.com/estore/browse/level_seven_software_products_only.jsp?productId=131414).

### Quality controls and analytical sequence

**Fig. 1:**
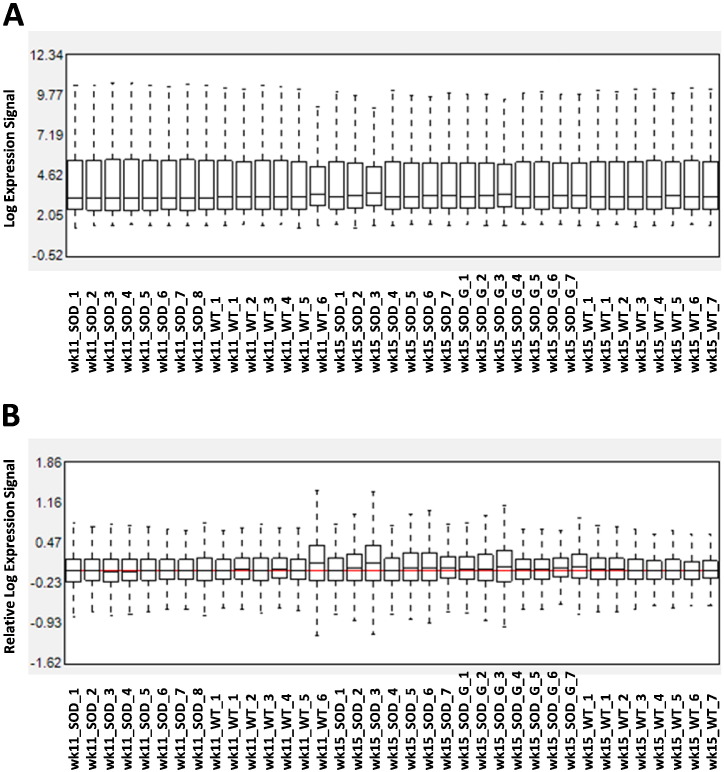
Expression signal normalization. Expression signal before (A) and after normalization (B) by Affymetrix Expression Console software. The red line in panel B shows the median of all arrays.

The efficacy of the intensity normalization was assessed with box plots showing probe cell intensity before normalization and relative probe cell intensity after normalization (Fig. 1A, B). After normalization, variability among arrays was strongly reduced, as monitored by the nearness between the median of log expression signals from each samples to the median array of all samples (red line, Fig. 1B).

Enrichment in mRNA from motoneurons was assessed by comparing the relative expression of cell specific genes [motoneurons: neurofilament-heavy chain (Nefh); astrocytes: glial fibrillary acidic protein (gfap); oligodendrocytes: SRY (sex determining region Y)-box 10(Sox10); microglia: allograft inflammatory factor 1-like (Aif1l); endothelial cells: claudin 5(Cldn5)] [5].

**Fig. 2:**
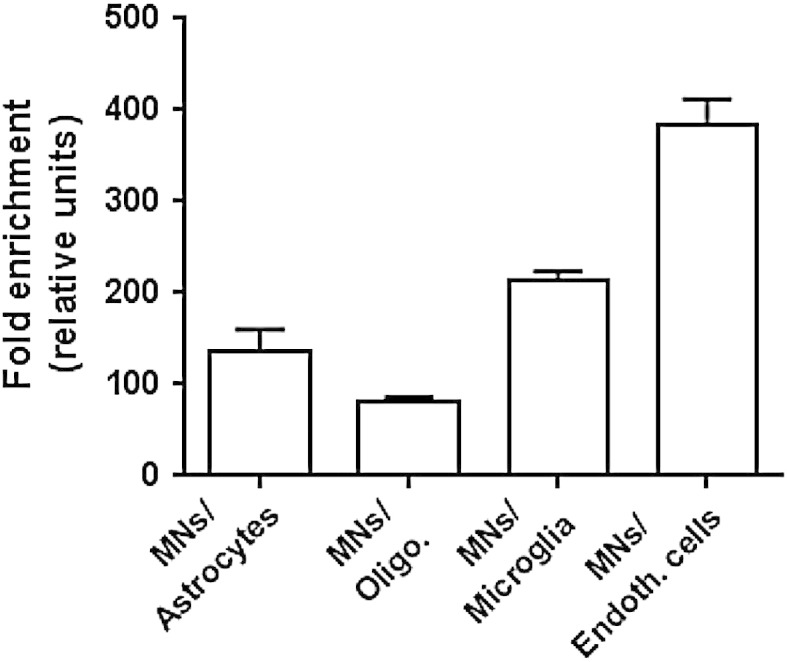
Relative fold enrichment in motoneuron mRNA. Given is the relative fold enrichment in mRNA from motoneurons, as determined by the ratio between neurofilament-heavy chain expression value and other genes specific for one cell types (GFAP for astrocytes; Sox10 for oligodendrocytes; Aif1l for microglial cells; Cldn5 for endothelial cells).

Based on the ratios between Nefh and the other cell specific transcripts, the laser microdissection led to an enrichment of motoneuronal mRNA estimated to a 135-fold as compared to astrocyte mRNA, to an 82-fold as compared to oligodendrocyte mRNA, to a 214-fold as compared to microglia mRNA and to a 384-fold as compared to endothelial mRNA (Fig. 2).

**Fig. 3:**
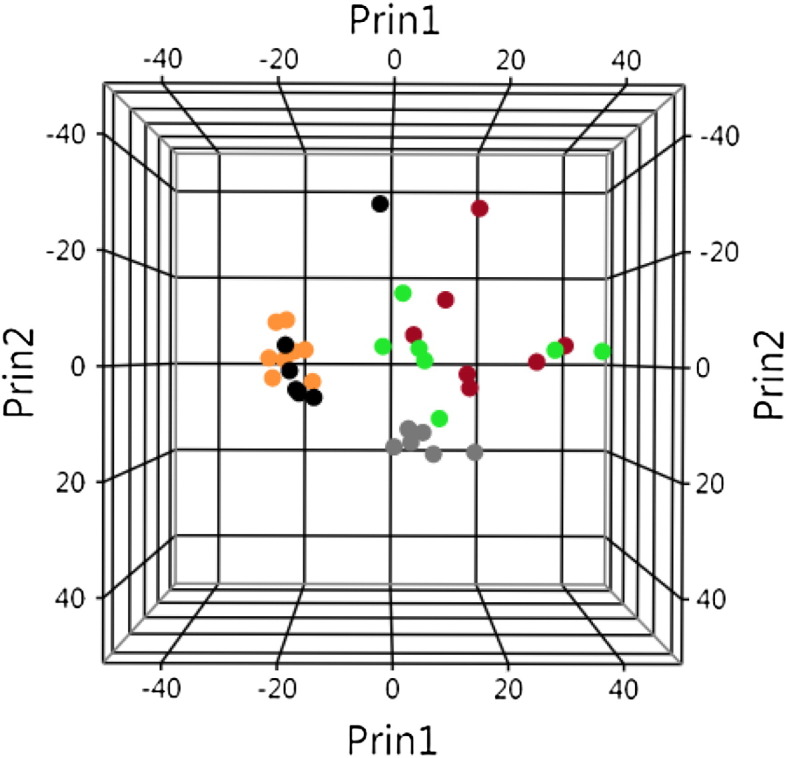
Principal component analysis. Given is the distribution of samples based on their global transcriptomic profiles. Non-transgenic mice are represented by black (11 weeks old) and gray (15 weeks old) dots. SOD1 G93A mice are represented by orange (11 weeks), red (15 weeks, placebo) and green (15 weeks, G-CSF treated) dots.

Principal component analysis (JMP 11.0.0, SAS Institute, Cary, North Carolina) was performed with the 1279 genes showing a standard deviation higher than 1.1 across all array to assess homogeneity among samples (Fig. 3). Age had a strong influence on the distribution of samples as shown by the segregation between samples from 11 weeks and 15 weeks old mice, regardless of the genotype.

At 15 weeks of age, samples from SOD1 G93A mice presented strong dispersion, presumably due to the variability of the pace of disease progression. It can be noted that five samples from the G-CSF treated mice regrouped together and were close to the non-transgenic or asymptomatic SOD1 G93A clusters. Samples from the placebo-treated SOD1 G93A mice presented the strongest dispersion. These data suggest that G-CSF had a substantial effect on the transcriptome of SOD1 G93A motoneurons.

Prior to supervised analysis, gene expression database was filtered for intensity signal (at least 4 arrays showed an intensity higher than centile 0.2) to remove background noise and for variability (standard deviation higher than centile 0.3) to discard transcripts with no or little changes in the expression across all arrays.

Discriminant analysis was used to determine how samples from SOD1 G93A mice treated with G-CSF relate to the other experimental groups. Only transcripts showing a standard deviation greater than 1.2 across all arrays were included in this analysis.

In an attempt to identify specific transcriptomic deregulation with biological relevance by ANOVA, we applied another filtering step, consisting in removing all transcripts with a fold-change expression lower than 1.2 when comparing non-transgenic and SOD1 G93A samples. The results generated by the discriminant analysis and by ANOVA are detailed in the original manuscript [1].
